# Pain relief that matters to patients: systematic review of empirical studies assessing the minimum clinically important difference in acute pain

**DOI:** 10.1186/s12916-016-0775-3

**Published:** 2017-02-20

**Authors:** Mette Frahm Olsen, Eik Bjerre, Maria Damkjær Hansen, Jørgen Hilden, Nino Emanuel Landler, Britta Tendal, Asbjørn Hróbjartsson

**Affiliations:** 1 Nordic Cochrane Centre, Rigshospitalet, Blegdamsvej 9, Department 7811, 2100 Copenhagen Ø, Denmark; 2 University Hospitals’ Centre for Health Research (UCSF), Rigshospitalet, Blegdamsvej 9, Department 9701, 2100 Copenhagen Ø, Denmark; 3 Frederiksberg Hospital, Nordre Fasanvej 57, 2000 Frederiksberg, Denmark; 4 Section of Biostatistics, University of Copenhagen, Østre Farigmagsgade 5, 114 Copenhagen Ø, Denmark; 5 Department of Cardiology, Herlev-Gentofte Hospital, Kildegårdsvej 28, 2900 Hellerup, Denmark; 6 Centre for Evidence-Based Medicine, University of Southern Denmark & Odense University Hospital, Sdr. Boulevard 29, Gate 50 (Videncenteret), 5000 Odense C, Denmark

**Keywords:** Pain, Minimum clinically important difference, Systematic review

## Abstract

**Background:**

The minimum clinically important difference (MCID) is used to interpret the clinical relevance of results reported by trials and meta-analyses as well as to plan sample sizes in new studies. However, there is a lack of consensus about the size of MCID in acute pain, which is a core symptom affecting patients across many clinical conditions.

**Methods:**

We identified and systematically reviewed empirical studies of MCID in acute pain. We searched PubMed, EMBASE and Cochrane Library, and included prospective studies determining MCID using a patient-reported anchor and a one-dimensional pain scale (e.g. 100 mm visual analogue scale). We summarised results and explored reasons for heterogeneity applying meta-regression, subgroup analyses and individual patient data meta-analyses.

**Results:**

We included 37 studies (8479 patients). Thirty-five studies used a mean change approach, i.e. MCID was assessed as the mean difference in pain score among patients who reported a minimum degree of improvement, while seven studies used a threshold approach, i.e. MCID was assessed as the threshold in pain reduction associated with the best accuracy (sensitivity and specificity) for identifying improved patients. Meta-analyses found considerable heterogeneity between studies (absolute MCID: I^2^ = 93%, relative MCID: I^2^ = 75%) and results were therefore presented qualitatively, while analyses focused on exploring reasons for heterogeneity. The reported absolute MCID values ranged widely from 8 to 40 mm (standardised to a 100 mm scale) and the relative MCID values from 13% to 85%. From analyses of individual patient data (seven studies, 918 patients), we found baseline pain strongly associated with absolute, but not relative, MCID as patients with higher baseline pain needed larger pain reduction to perceive relief. Subgroup analyses showed that the definition of improved patients (one or several categories improvement or meaningful change) and the design of studies (single or multiple measurements) also influenced MCID values.

**Conclusions:**

The MCID in acute pain varied greatly between studies and was influenced by baseline pain, definitions of improved patients and study design. MCID is context-specific and potentially misguiding if determined, applied or interpreted inappropriately. Explicit and conscientious reflections on the choice of a reference value are required when using MCID to classify research results as clinically important or trivial.

## Background

It can be challenging to decide whether a modest effect in a randomised clinical trial, or a meta-analysis of several such trials, is clinically relevant. Statistical tests inform on the probability of a result being a chance finding; however, they convey no information on whether a given effect will be experienced as important by patients. The degree of pain reduction that is considered clinically relevant has an impact on which analgesic interventions are regarded clinically useful. This interpretation problem for clinical relevance has been at the core of debates of the importance of several types of interventions intended for reducing acute pain, for example, paracetamol [1–3], non-steroidal anti-inflammatory drugs [4, 5], morphine or synthetic opiates [6], corticosteroids [7], muscle relaxants [4], laser therapy [8], transcranial direct current stimulation [9], EMLA cream [10], and acupuncture [11]. A related challenge involves the calculation of sample sizes for clinical trials, where researchers need to know the smallest clinically important effect that the trial should not miss to be able to determine an adequate sample size.

Jaeschke et al. [12] characterised the concept of minimum clinically relevant difference in 1989 as “*the smallest difference in score in the domain of interest which participants perceive as beneficial and which would mandate, in the absence of troublesome side effects and costs, a change in the patient’s management*”. The strength of the concept is that it defines a relevant effect size based on clinical considerations and not only statistical significance [13, 14]. It has subsequently been supplemented by a related concept – the substantial (and not only minimum) clinically relevant difference [15].

The minimum clinically important difference (MCID) is sometimes chosen on the basis of expert consensus judgement [16], statistical models [17] or objective criteria [18]. However, in acute pain, it seems reasonable to anchor clinical relevance to the patients’ experience. This approach is in accordance with the increasing awareness of the relevance of patient-reported outcomes in clinical research [19]. Several such empirical studies have been conducted to determine the MCID in acute pain, but they differ with regard to methodology, clinical condition and findings, and have not yet been systematically reviewed. Since acute pain is a core symptom in healthcare, an assessment of the MCID and a clarification of the causes for its variation will have broad interest. It has been suggested that baseline pain influences absolute values of MCID, but study reports have been conflicting [20, 21], and it remains unclear which other clinical or methodological factors are of importance.

We therefore decided to systematically review empirical studies of the MCID in acute pain relief and to examine possible causes for variation between study results, especially their likely dependency on baseline pain levels. We also reviewed studies of the substantial clinically important difference in acute pain relief as well as clinically important differences for worsening of pain.

## Methods

### Eligibility criteria

We included prospective studies of patients with acute pain, regardless of age, clinical condition, and intervention, in which pain intensity was assessed on a one-dimensional scale, e.g. a 100 mm visual analogue scale (VAS) or a 0–10 point numerical rating scale (NRS), and in which the MCID was determined using an anchor-based method using patients’ perception of change to determine clinical importance. Pain was considered acute when its duration was less than 1 month or, if duration was not indicated, when it was described as such in a study report.

Studies were excluded if they were not clinical (i.e. used healthy volunteers) or determined the MCID from objective criteria (e.g. return to work), the distribution of data (e.g. the minimum detectable difference) or expert consensus.

A typical eligible study would ask patients to score their pain intensity, e.g. using a VAS, at baseline and follow-up. At follow-up, patients were also asked to categorise their change in pain intensity using response options such as ‘no change’, ‘a little better’/‘somewhat better’, and ‘a lot better’/‘much better’. The MCID was then determined from the change in scores on the pain scale among patients having categorised their change as ‘a little better’ (or a similar expression indicating a minimum clinically important improvement).

We included studies with two types of analytical approaches (1) the ‘mean change approach’, i.e. the mean difference in pain scores among patients who reported a minimum degree of pain relief [22]; or (2) the ‘threshold approach’, i.e. the threshold value for pain score change which most accurately (yield﻿i﻿ng best sensitivity and specificity) identified patients experiencing relevant pain relief in analogy with a diagnostic test where the gold standard is patients’ perception of change [23].

### Search strategy

We searched PubMed, EMBASE and Cochrane Library until August 2016 with no language restrictions. The core search string was: (minimal OR minimally OR minimum OR ‘clinically significant’ OR ‘clinically important’ OR ‘clinically meaningful’ OR ‘clinically relevant’) AND (difference OR change OR relief OR reduction) AND (‘pain measurement*’ OR ‘visual analog scale’ OR ‘numeric rating scale’) AND (pain) with variations according to the specific database (Appendix 1). The reference lists of all included studies and relevant review papers were read systematically to identify further studies.

Screening of titles and abstracts to determine the eligibility of studies was done by the primary author (MFO), while the selected full-text records were examined by two researchers independently (MFO and either EB, NEL, BT, or MDH). Any disagreement was solved by discussion.

### Data extraction and retrieval

Data extraction was conducted by two researchers independently (MFO and EB, BT, or NEL) using pretested data extraction forms generated in EpiData (EpiData Association, Odense, Denmark). Any disagreements were solved by discussion.

For each study, we extracted descriptive data including publication year, study design, setting, clinical condition, type of intervention, sampling method, sample size, and definition of patients with relevant change (see Appendix 2 for complete list). For studies using a mean change approach, we extracted the following outcome data: the MCID for pain relief (absolute values in mm or points and relative value in percent change from baseline) and for pain worsening (absolute and relative values), as well as the substantial clinically important difference for relief and worsening of pain (absolute and relative values). We extracted MCIDs as the mean change in pain score among patients who indicated a one-category improvement (e.g. ‘a little better’). If unavailable, we extracted the mean change among patients who were minimally improved by authors’ definition (e.g. some authors defined minimum important change as the mean change in pain score among patients with either a one- or two-category improvement). Similarly, we extracted the substantial clinically important differences as the mean change among patients with a two-category improvement or used the authors’ definition. We extracted the point estimate of outcomes with their corresponding standard error or, if unavailable, other measures of variation such as standard deviation or 95% confidence interval.

For studies using a threshold approach, we extracted information about definition of responders (i.e. patients with relevant change) and non-responders and the cut-off point with its corresponding sensitivity (i.e. percentage of responders correctly classified as such) and specificity (i.e. percentage of non-responders correctly classified as such). If studies reported pain scores from several concurrent pain assessments (e.g. back pain and leg pain), we extracted the assessment where more data were available or, if no difference found, we randomly selected which to extract. All scales were standardised to a 0–100 mm scale. When studies reported pain assessments based on both VAS and NRS, we used the assessment based on VAS.

If the primary outcome or other key variables were unclear or incompletely reported from a study, we contacted the corresponding author. In cases where authors provided individual patient data, we first checked whether we could replicate a main result of the published paper. We then calculated estimates of absolute and relative MCIDs and their corresponding standard error.

For each study, we assessed risk of attrition bias (studies were considered low risk when attrition < 10%) and risk of non-representative study sample (studies were considered low risk if using consecutive or random sampling).

### Data synthesis and analysis

For each study, we extracted or calculated the MCID for pain relief (absolute and relative change), and noted results of any study-based exploration of causes for variation, e.g. baseline pain.

We then summarised results qualitatively as there was considerable clinical and methodological variation between studies and heterogeneity in their results. To provide an overview, we first reported the range of results for all studies and then the range and median results with inter-quartile ranges (IQR) of studies according to analytical strategy (mean change or threshold approach). To facilitate exploration of reasons for heterogeneity, we then pooled results of studies using mean change approach with inverse-variance meta-analysis using random effects models. We studied the association with baseline pain scores in three different analyses. First, we explored the impact of the average population baseline pain in a mixed-effects meta-regression (acknowledging the limitations of aggregated data-level analysis [24]). Second, we pooled individual patient data to estimate the MCID (absolute and relative changes) using a two-stage individual participant data meta-analysis. In these models, MCIDs were first estimated in each individual study using a mixed model based on all repeated measurements and participant specific random effects to capture the serial correlation within each participant. Results were then pooled using random-effects inverse-variance meta-analysis. Third, we used the same model to derive outcomes measured at different time points in studies with multiple measurements per patients (i.e. the use of a ‘moving baseline’).

We furthermore used subgroup analyses to explore whether between-study variation was explained by differences in other clinical and methodological factors including clinical condition, type of pain scale (VAS vs. NRS), directionality of global transition scale (one-sided vs. two-sided scale), definition of minimum clinically important change (one category vs. several categories improvement vs. distinction between meaningful and non-meaningful change), number of pain assessments per patients (single vs. multiple assessments adjusted for correlation vs. multiple unadjusted assessments), risk of attrition bias (low vs. high or unclear) and risk of non-representative sample (low vs. high or unclear).

Finally, we used the same analysis strategy for the substantial clinically important difference for pain relief (absolute and relative change) and the minimum and substantial clinically important differences for worsening of pain (absolute and relative change).

All data analyses were done using Stata/IC version 13.

## Results

### Study selection

**Fig. 1:**
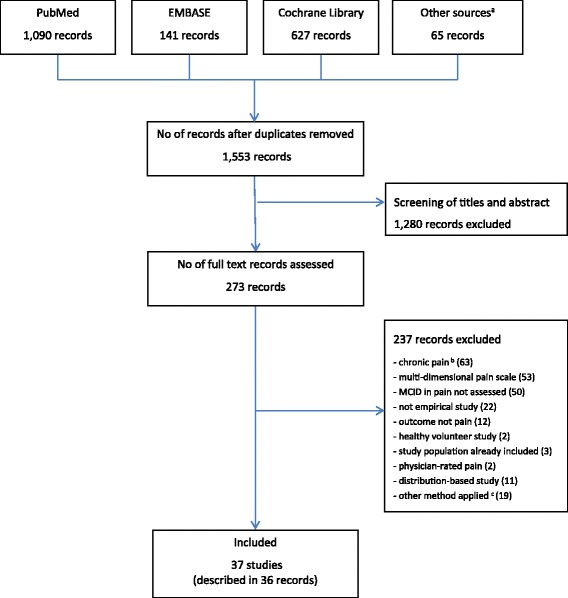
Flow chart of study identification. MCID = Minimal Clinically Important Difference, ^a^Additional records identified through “related papers” function in databases and reference lists of screened papers, ^b^Two records include both acute and chronic pain studies, ^c^Includes studies in which MCID was not based on subjective patient-reported assessment of pain relief

We screened 1553 database records and read 273 full-text publications (Fig. 1). We excluded 237 publications, mostly because they included patients with chronic pain (n = 63) or multi-dimensional pain scales (n = 53). Thus, we included 36 publications [21, 25–59] reporting 37 studies (8479 patients).

### Characteristics of included studies

The majority of studies were based in emergency departments and included a mix of patients with acute pain of both traumatic and non-traumatic origin (10 studies) or unspecified pain (7 studies). Additional studies included patients with post-operative pain, cancer-related pain, sickle cell crisis, rheumatic pain, abdominal pain, low back pain, or headache (Tables 1 and 2). All studies were published in English.

**Table 1:** Studies determining minimal clinically important difference as a mean change^a^ (35 studies, 7894 patients)

Study	n	Patient characteristics	Methodological characteristics							MCID	
		Clinical condition	Age, years (range)^b^	Male sex, %	Baseline pain, mm	Pain scale	Transition scale (directionality, categories)	Follow-up (Analysis adjusted)^c^	Category defining MCID	Absolute^d^ mm (95% CI)	Relative^e^ % (95% CI)
Aicher [25]	1743	Headache	38^h^ (16–72)	24	64	VAS	1-sided, 4 cat	Single	Less good efficacy	27 (25–29)	NR
Bailey [26]	202	Mixed^g^	12 (8–17)	57	NR	NRS	2-sided, 5 cat	Single	A little better	10 (0–20)^h^	NR
Barton 2002 (reported in Jensen 2002 [43])	125	Post-operation pain (laparotomy)	41	0	NR	VAS	1-sided, 5 cat	Multiple (unclear)	A little relief	9 (7–12)	13 (8–17)
Bernstein [27]	135	Unspecified	42 (21–65)	45	100^h^	NRS	1-sided, 5 cat	Single	A little relief	21 (18–24)	NR
Bijur [28]^f^	108	Unspecified	44 (21–85)	40	73	VAS + NRS	2-sided, 5 cat	Multiple (no)	A little less pain	16 (11–21)	22 (15–28)
Bijur [29]^f^	195	Unspecified	74	27	74	NRS	2-sided, 5 cat	Multiple (yes)	A little less pain	15 (14–17)	24 (21–27)
Bijur [30]^f^	214	Unspecified	74	34	86	NRS	2-sided, 5 cat	Multiple (yes)	A little less pain	14 (12–15)	22 (19–25)
Bird [31]	77	Isolated extremity trauma	38	62	52	VAS	2-sided, 5 cat	Multiple (no)	A little less pain	20 (15–25)	NR
Bulloch [32]	121	Mixed^g^ (65% traumatic, 35% non-traumatic)	10 (5–16)	56	60^h^	VAS	2-sided, 5 cat	Multiple (no)	A little less pain	17 (11–23)	NR
Cepeda [33]	700	Post-operation pain (mixed)	41 (16–88)	38	79	NRS	1-sided, 5 cat	Multiple (yes)	Minimal improvement	17 (10–20)^h^	20 (13–30)^h^
Chow [34]	105	Pain flare after radiotherapy	70^h^	56	50	NRS	2-sided, 3 cat	Multiple (no)	Better	23 (20–26)	NR
Degerli [35]	306	Thoraco-abdominal pain (non-traumatic)	42 (17–83)	40	67	VAS	2-sided, 5 cat	Multiple (no)	A little less pain	25 (23–26)	NR
Farrar [36]	134	Cancer-related breakthrough pain	55 (21–87)	53	NR	NRS	1-sided, 5 cat	Multiple (yes)	Slight relief	13–19^j^	16–34^j^
Fosnocht [37]	1490	Mixed^g^	36 (18–89)	47	63	VAS	2-sided, 5 cat	Multiple (no)	A little less pain	21 (19–23)	NR
Gallagher [38]^f^	96	Mixed^g^	37 (19–71)	45	64	VAS	2-sided, 5 cat	Multiple (yes)	A little less pain	15 (11–18)	17 (6–29)
Gallagher [39]^f^	101	Abdominal pain	40 (15–88)	39	65	VAS	2-sided, 5 cat	Multiple (yes)	A little less pain	18 (14–21)	23 (12–33)
Grilo [40]	50	Rheumatic conditions	49 (16–85)	44	78	VAS	1-sided, 5 cat	Single	Moderate relief	40^i^	NR
Grotle [41]	54	Acute low back pain	38	27	39	VAS + NRS	2-sided, 6 cat	Single	Slightly improved	34 (20–48)	
Holdgate [42]^f^	79	Unspecified	53 (21–93)	NR	54	VAS + NRS	2-sided, 5 cat	Multiple (yes)	A bit better	16 (12–20)	34 (27–41)
Kelly [44] Kelly [45, 46]	156	Mixed^g^ (43% traumatic, 57% non-traumatic)	44 (16–97)	56	51	VAS	2-sided, 5 cat	Multiple (unclear)	A little better	8 (4–12)	NR
Kendrick 2005 [47]	356	Mixed^g^ (35% traumatic, 65% non-traumatic)	41^h^	42	66	NRS	2-sided, 5 cat	Multiple (no)	A little less pain	15 (13–16)	NR
Lopez [21]	74	Sickle cell crisis	NR	NR	79	VAS	2-sided, 5 cat	Multiple (no)	A little better	14 (11–16)^h,k^	NR
Mark [48]	186	Unspecified	49 (15–87)	38	65	VAS	2-sided, 5 cat	Single	A little better	17 (14–20)^k^	NR
Martin [49]	95	Third molar extraction	30	40	33	VAS	2-sided, 7 cat	Single	Much improved or best ever	23^i^	72^i^
Martin [49]	63	Third molar extraction	25	40	35	Do	Do	Do	Do	25^i^	69^i^
Martin [49]	62	Third molar extraction	27	50	30	Do	Do	Do	Do	29^i^	85^i^
McConahay [50]	126	Mixed^g^ (48% traumatic, 52% non-traumatic)	9 (5–12)	56	83	VAS	2-sided, 5 cat	Single	A little less pain	24 (17–31)	NR
Mohan [51]^f^	125	Unspecified	35^h^	44	46	VAS + NRS	2-sided, 5 cat	Multiple (yes)	A bit better	13 (9–17)	18 (1–35)
Myrvik [52]	28	Sickle cell crisis	15 (8–18)	50	75^h^	VAS + NRS	2-sided, 5 cat	Multiple (unclear)	A little better	10 (4–15)	NR
Powell [53]	73	Mixed (63% traumatic, 37% non-traumatic)	12 (8–15)	NR	60	VAS	2-sided, 5 cat	Multiple (yes)	A bit better	11^i^	NR
Rasmussen 2002 (reported in Jensen 2002 [43])	123	Post-operation pain (knee surgery)	65	34	NR	VAS	1-sided, 5 cat	Multiple (unclear)	A little relief	13 (10–17)	18 (14–22)
Sloman [54]	150	Post-operation pain (mixed)	47 (14–89)	53	67	NRS	1-sided, 5 cat	Single	Minimal relief	18^i^	36 (21–51)
Stahmer [55]	81	Mixed^g^ (31% traumatic, 69% non-traumatic)	38 (16–87)	44	68	NRS	1-sided, 5 cat	Single	Some relief or partial relief	NR	30 (18–42)
Todd [56]	48	Trauma	35^h^ (17–76)	69	56	VAS	2-sided, 5 cat	Multiple (no)	A little less pain	16 (10–22)	NR
Voepel-Lewis [59]	113	Post-operation pain (e.g. spinal fusion, splenectomy)	13 (7–16)	43	NR	NRS	2-sided, 5 cat	Multiple (yes)	A little better	10 (5–15)	NR

^a^Mean change in pain score among patients with minimum improvement of pain

^b^Mean (range) if not otherwise indicated,

^c^Estimate based on single or multiple follow-up measurements (were standard errors adjusted for dependency between multiple measurements?)

^d^Absolute = mm reduction on a 100 mm scale

^e^Relative = % reduction from baseline

^f^Individual patient data provided by authors

^g^Mixed patient population recruited at Hospital Emergency Department

^h^Data is median (inter-quartile range)

^i^95% CI unavailable

^j^Estimates for subgroups with different baseline pain

^k^Patients with minimal improvement and worsening of pain were combined

*MCID* minimum clinically important difference, *VAS* visual analogue scale, *NRS* numeric rating scale, *CI* confidence interval, *NR* not reported

**Table 2:** Studies determining minimal clinically important difference as a threshold value^a^ (7 studies, 2602 patients)

Study	n	Patient characteristics				Methodological characteristics				MCID	
		Clinical condition	Age, years^b^	Male sex, %	Baseline pain, mm	Pain scale	Transition scale (directionality, categories)	Anchor question	Categories defining responders	Absolute, mm (sensitivity, specificity)	Relative, % (sensitivity, specificity)
Aicher [25]	1743	Headache	38^b^ (16–72)	24	64	VAS	1-sided, 4 cat (0 to +4 points)	"How do you assess the efficacy of your tablets?"	Good (+3) or very good (+4)	35 (76%, 76%)	NR
Grotle [41]	54	Acute low back pain	38	27	39	VAS + NRS	2-sided, 6 cat (–1 to +4 points)	"To which extent has your back problem changed?"	Slightly improved (+1), much better (+2), very much better (+3), or completely gone (+4)	10 (87%, 81%)	NR
Martin [49]	63	Post-operation pain (third molar extraction)	30	40	33	VAS	2-sided, 7 cat (–3 to +3 points)	Assessment of recovery following treatment	Much improved (+2) or best ever (+3)	10 (70%, 82%)	50 (82%, 82%)
Martin [49]	62	Post-operation pain (third molar extraction)	25	40	35	Do	Do	Do	Do	10 (71%, 71%)	50 (83%, 86%)
Martin [49]	95	Post-operation pain (third molar extraction)	27	50	30	Do	Do	Do	Do	10 (87%, 69%)	50 (97%, 82%)
Tsze [57]	314	Mixed^d^	10	50	NR	VAS	2-sided, 5 cat (–2 to +2 points)	Assessment of change in pain following treatment	A little better (+1)	10 (76%, 74%)	15 (78%, 79%)
Tubach [58]	271	Rotator cuff syndrome	48	38	68	NRS	2-sided, 15 cat (–7 to +7 points)	Assessment of response to treatment	A good deal better (+5) or a great deal better (+6)	34–63^c^ (75%, NR)	NR

*MCID* minimum clinically important difference (mm change on a 100 mm scale), *VAS* visual analogue scale, *NRS* numeric rating scale, *NR* not reported

^a^Threshold in pain reduction associated with the best accuracy (sensitivity and specificity) for identifying improved patients

^b^Data is median (range)

^c^Estimates for subgroups with different baseline pain

^c^Mixed patient population recruited at Hospital Emergency Department

Twenty studies assessed pain using a 100 mm VAS (or the similar Color Analog Scale), and 12 studies used an 11-point NRS (0–10), while five studies used both scales. In 32 studies, patients compared their current pain intensity with pain at their previous assessment, while they were asked to assess the effect of their treatment in five studies [25, 49, 58]. Transition scales were either two-sided (29 studies) with 3–15 response categories for both improvement and deterioration, or one-sided (8 studies) with five response categories addressing only improvement.

For studies using the mean change approach, the majority defined the minimum clinically important improvement as a one-category improvement on the transition scale (31 studies). The response categories were similar with wordings such as ‘a little less pain’, ‘a bit better’, ‘slightly improved’, or ‘slight relief’. In four studies, the MCID was defined as the mean change in pain score among patients with a one- or two-category improvement, thereby combing patients answering ‘some relief’ and ‘partial relief’ [55], or ‘much improved’ and ‘best ever’ [49–51]. Finally, two studies differentiated between non-important and important change, using the categories ‘inadequate relief’ and ‘moderate relief’ in one study [40], and ‘poor’ and ‘less good’ efficiency of treatment in another [25] (Table 1). Studies using the threshold approach had large variation in transition scales and definitions of responders versus non-responders; patients were regarded as importantly improved if they indicated a one-category relief in two studies [41, 57], while they needed a five-category improvement in another [58] (Table 2).

Pain intensity was assessed at baseline and a single follow-up measurement in 14 studies, while it was assessed at multiple (from 2 to 16) follow-ups with intervals between 10 and 45 minutes in 23 studies. The latter group then derived their outcome as the summarised mean difference in pain score from the patients’ previous pain assessment when they reported minimum relief (i.e. a series of ‘moving baselines’). In eight of these studies, the *P* values of analyses were adjusted for correlation between estimates, for example, with Generalised Estimation Equation, while the remaining studies either made no adjustment or did not report this. Access to individual patient data increased the number of studies with adjusted estimates to 11.

In 10 studies, the MCID was defined as a numeric change for all patients with minimum change, regardless of whether pain had improved or worsened. After contacting authors, separate estimates for pain relief were available from eight of these.

### MCID regardless of analytical approach

Standardised to a 100-mm scale, the absolute MCID in 30 studies ranged from 8 to 40 mm, and the relative difference in 15 studies ranged from 13% to 85%.

### MCIDs in studies using the mean change approach

**Fig. 2:**
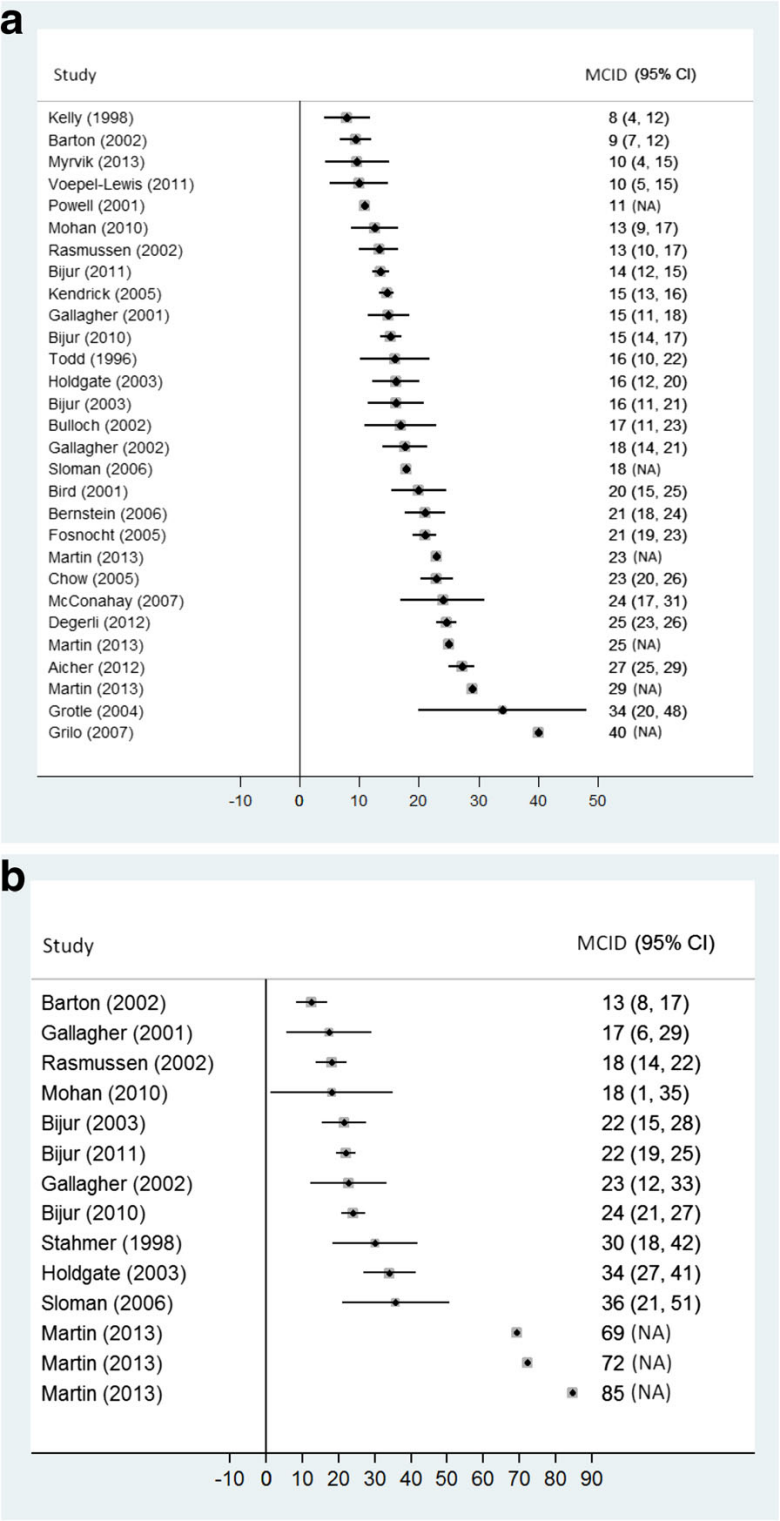
**a** Range of absolute minimal clinically important differences in acute pain assessed by the mean change approach* (29 studies, 6517 patients). *MCID assessed as the mean change in pain score among patients with minimal improvement of pain, MCID = Minimal Clinically Important Difference (mm reduction on a 100 mm scale), Studies where standard error (or data for obtaining this) was unavailable are presented as point estimates without 95% CI (NA = not applicable). **b** Range of relative minimal clinically important differences in acute pain assessed by the mean change approach* (14 studies, 1617 patients). *MCID assessed as the mean change in pain score among patients with minimal improvement of pain, MCID = Minimal Clinically Important Difference (% reduction from baseline), Studies where standard error (or data for obtaining this) was unavailable are presented as point estimates without 95% CI (NA = not applicable)

The determination of the MCID was based on a mean change approach in 35 studies, of which 30 (6598 patients) were included in our analyses and five were disregarded (see below). Twenty-nine studies (6517 patients) reported absolute values ranging from 8 to 40 mm, with a median of 17 mm (IQR 14–23 mm) (Fig. 2a). Only nine of the 30 studies reported relative MCIDs, but access to individual patient data made relative values available from 14 studies (1617 patients) ranging from 13% to 85%, with a median of 23% (IQR 18–36%) (Fig. 2b).

For data syntheses, we did not include results from five of the 35 studies (1567 patients) as they did not differentiate between pain relief and pain worsening [21, 48], because median (and not mean) differences in pain were reported [26, 33], or because outcomes were reported for subgroups and no overall estimate could be derived [36]. The range of MCID in these studies was comparable to the included studies: 10–19 mm. An additional six studies (493 patients) were not included in meta-analysis since information about standard error of estimates was unavailable [40, 49, 53, 54]. The results from these studies ranged from 11 to 40 mm.

We had data usable for meta-analyses from 23 studies (6024 patients) reporting absolute values and 11 studies (1397 patients) reporting relative values. Meta-analyses of both the absolute and relative values showed considerable heterogeneity: I^2^ = 93%, *P* < 0.001 and I^2^ = 75%, *P* < 0.001 (Table 3). We present the meta-analyses for completeness and as a basis for exploring reasons for the heterogeneity, but stress that medians and interquartile ranges are more appropriate descriptors of the results.

**Table 3:** Clinically important differences in acute pain

		Analysis of medians^a^		Analysis of pooled average	
Clinically important difference	Range	Number of studies (patients^b^)	MCID median (IQR)	Number of studies (patients^b^)	MCID pooled average (95% CI), I^2 c^
Minimum clinically important difference for pain relief					
Absolute change, mm					
Mean change approach	8 to 40	29 (6517)	17 (14 to 23)	23 (6024)	17 (15 to 19), 93%
Threshold approach	10 to 35	6 (2331)	10 (10 to 10)	NA	NA
Relative change, %					
Mean change approach	13 to 85	14 (1617)	23 (18 to 36)	11 (1397)	22 (19 to 26), 75%
Threshold approach	15 to 50	4 (534)	50 (33 to 50)	NA	NA
Substantial clinically important difference for pain relief					
Absolute change, mm					
Mean change approach	18 to 54	23 (6114)	32 (24 to 38)	21 (5891)	32 (27 to 38), 97%
Relative change, %					
Mean change approach	36 to 78	11 (1397)	57 (45 to 65)	11 (1397)	57 (47 to 67), 94%
Minimum clinically important difference for pain worsening					
Absolute change, mm					
Mean change approach	−21 to –8	18 (3822)	−11 (–13 to –10)	16 (3644)	−12 (–14 to –11), 62%
Relative change, %					
Mean change approach	−89 to –17	7 (918)	−44 (–90 to –16)	7 (918)	−35 (–47 to –23), 67%
Substantial clinically important difference for pain worsening					
Absolute change, mm					
Mean change approach	−66 to 0	16 (3663)	−21 (–28 to –16)	14 (3464)	−24 (–29 to –18), 71%
Relative change, %					
Mean change approach	−292 to –18	7 (918)	−83 (–292 to –18)	7 (918)	−34 (–49 to –19), 20%

*MCID* minimum clinically important difference (mm or % reduction on a 100 mm scale), *SCID* substantial clinically important difference (mm or % reduction on a 100 mm scale), *IQR* inter-quartile range, *NA* not applicable

^a^The median is based on studies included in the pooled average as well as studies with unavailable standard errors

^b^Total number of patients in the included studies

^c^I^2^ is the percentage of the variability in results that is due to heterogeneity rather than sampling error (chance); I^2^ of 0% to 40% might not be important, 30% to 60% may represent moderate heterogeneity, 50% to 90% may represent substantial heterogeneity, and 75% to 100% represents considerable heterogeneity

### MCID in studies using the threshold approach

Seven of the 37 included studies (2602 patients) determined clinically important differences as a threshold to differentiate between patients with or without relevant pain relief. Absolute thresholds ranged from 10 to 35 mm in six studies (2331 patients) with a median of 10 mm, and the relative threshold ranged from 15% to 50% in four studies (534 patients) (Table 3). In one additional study [58], patients were defined as responders if they indicated at least a five-category improvement. The corresponding clinically important differences were thus higher (34 to 63 mm depending on baseline pain) than in studies where patients only needed a one- [41, 57], two- [49], or three-category improvement [25], respectively, to be defined as responders (Table 2).

### Impact of baseline pain scores on the MCID

Eleven studies had assessed the possible influence of baseline pain on minimum clinically improvement (Appendix 3). Of nine studies assessing absolute change, seven reported an association [31, 33, 36, 43, 58, 59]. The two remaining studies found no association, but these were disregarded since they determined the MCID without differentiating between pain relief and pain worsening [21, 45]. Six studies assessed the association between baseline pain and relative change and either found the association to be non-significant or found it to be weaker than for absolute change.

Based on meta-regression, we found no association between baseline pain and either absolute (20 studies, *P* = 0.70) nor relative (9 studies, *P* = 0.83) estimates of MCIDs.

**Fig. 3:**
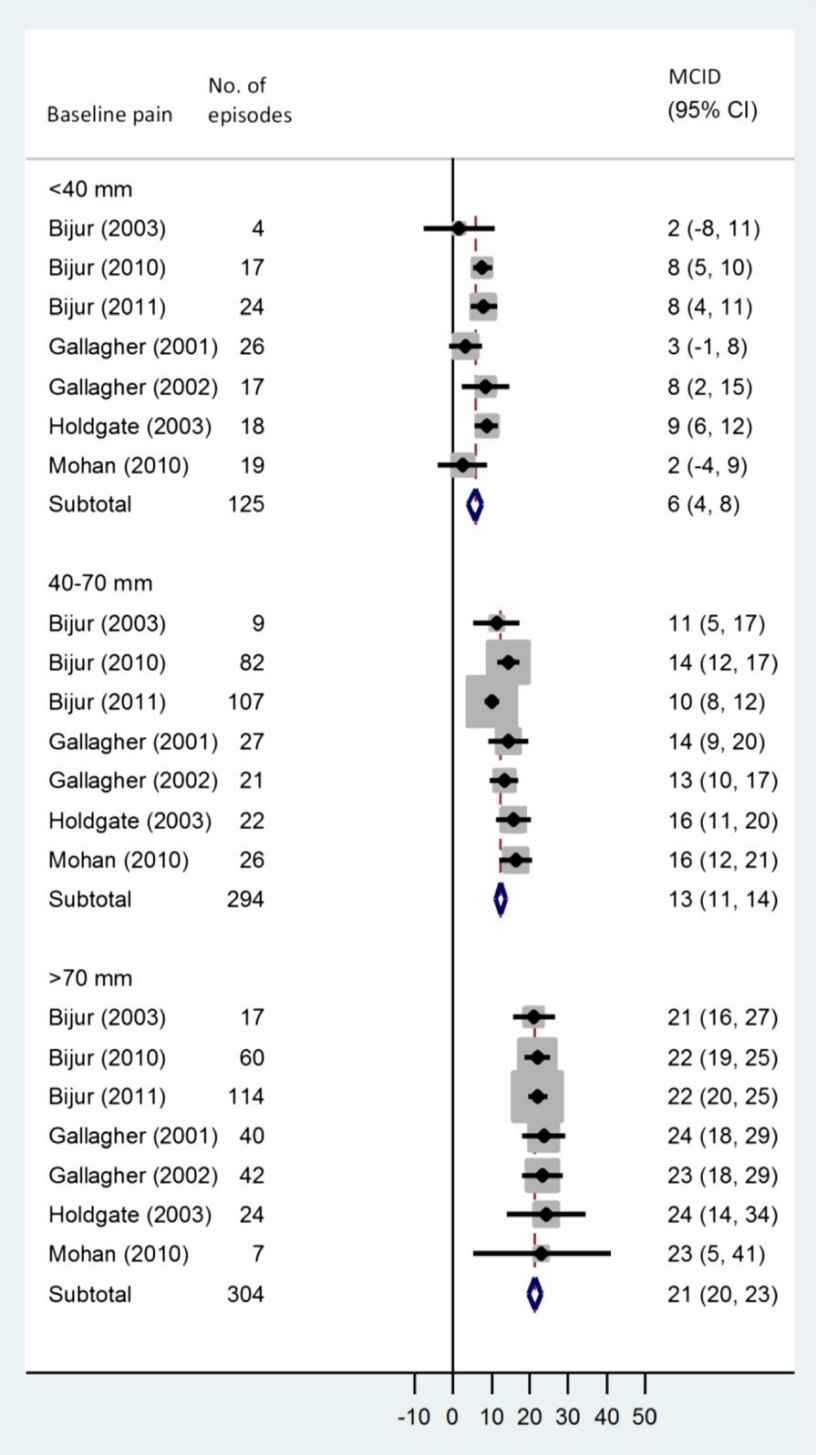
Minimum clinically important difference by baseline pain (individual patient data from 7 studies, 918 patients). MCID assessed as the mean change in pain score among patients with minimal improvement of pain, MCID = Minimal Clinically Important Difference (mm reduction on a 100 mm scale). Forest plot of mean pain difference in 723 episodes where patients reported pain to be “a little better” in 7 studies including a total of 918 patients

However, based on individual patient data meta-analysis from seven studies (918 patients), we found a strong association between baseline pain and absolute MCID, showing that patients with higher baseline pain need larger pain reduction to reach a minimum clinically relevant difference compared to patients with lower baseline pain. For each 10 mm increase in baseline pain, MCID increased by 3.1 mm (95% confidence interval, 2.8–3.5 mm, *P* < 0.001, I^2^ = 0%). Thus, for patients with initial pain levels < 40, 40–70, and > 70 mm, the MCIDs were 6 (4–8) mm, 13 (11–14) mm, and 21 (20–23) mm, respectively (Fig. 3). The corresponding relative estimates of MCIDs did not differ significantly: 17 (4–30) %, 23 (20–25) %, and 24 (22–26) %, respectively. In contrast, we found that patients with more severe pain perceived worsening of their status with smaller increases of pain intensity: the absolute MCID was 2.3 mm less for each 10 mm increase in baseline pain (1.4–3.0, *P* < 0.001, I^2^ = 67%).

From individual patient data, we also found that the MCID decreased with increasing time from baseline, from 17 (12 to 21) mm at 30 minutes to 11 (8 to 14) mm at 120 minutes. However, patients’ pain level declined accordingly during the multiple follow-ups and estimates expressed as a relative change from the previous assessment therefore did not decline.

### Impact of other clinical and methodological factors

Other causes for variation in the MCID were addressed in 10 studies. These included sex [33, 35, 36, 44–48, 51, 54, 57, 59], age [33, 35, 36, 44–46, 48, 51, 54, 57, 59], education level [35, 48, 51, 54], ethnicity [54, 57], primary language (English or Spanish) [57], and religion [54], as well as cause (traumatic or non-traumatic) [44–48, 57], duration [35] and location [35] of pain. None of the studies found associations except for one [48], which reported higher MCIDs for men compared to women and for non-traumatic pain compared to traumatic pain. However, these analyses were not adjusted for differences in baseline pain.

The MCIDs of subgroups are presented as medians and pooled averages, respectively (Table 4). For most clinical and methodological factors, the number of studies in each subgroup was too small to ensure detection of relevant differences between them. Nevertheless, although only few studies defined the MCID as the mean pain reduction among patients with several categories of improvement, or patients with ‘meaningful’ (and not just ‘minimum’) change, it was clear that these studies found higher MCIDs (medians 25 (IQR 23–29) and 34 (IQR 27–40), respectively) than studies where it was defined as the mean pain reduction among patients with a one-category improvement (median 16 (IQR 13–21)). It was also clear that the MCID was higher when based on a single assessment involving one fixed baseline value (median 25 (IQR 23–29)), than when summarised from multiple assessments with the previous assessment applied as a ‘moving baseline’ (medians 15 (IQR 13–16) and 16 (IQR 10–21), respectively). Subgroups of studies among patients with various clinical conditions were underpowered to detect relevant differences. The comparison of one- and two-sided transition scales was also underpowered, but the difference in scales did not seem to affect study results, while the comparison of VAS and NRS included enough studies to conclude that type of pain scale did not influence the MCID. Finally, we did not find differences in outcomes relating to the risk of attrition bias or risk of non-representative samples.

**Table 4:** Subgroup analyses of absolute minimum clinically important differences (MCIDs) for pain relief

Subgroup	Analysis of medians			Analysis of pooled average	
	Range, mm	Number of studies^a^ (patients^b^)	MCID Median, mm (IQR)	Number of studies (patients^b^)	MCID pooled average, mm (95% CI), I^2 c^
All studies	8–40	29 (6517)	17 (14–23)	23 (6024)	17 (15–19), 93%
Clinical conditions					
Post-operation pain^d^	9–29	7 (731)	18 (10–25)	3 (361)	11 (8–14), 42%
Trauma	16–20	2 (125)	18 (16–20)	2 (125)	18 (15–22), 11%
Abdominal pain	18–25	2 (407)	21 (18–25)	2 (407)	21 (15–28), 91%
Mixed patients at ED	8–24	7 (2418)	15 (11–21)	6 (2345)	16 (12–20), 90%
Other^e^	10–40	5 (1980)	27 (23–34)	4 (1930)	22 (15–29), 92%
Unspecified	13–21	6 (856)	16 (14–16)	6 (856)	16 (14–18), 72%
Definition of MCID					
One category improvement	8–34	24 (4504)	16 (13–21)	22 (4281)	16 (14–18), 91%
Several categories improvement	23–29	3 (220)	25 (23–29)	NA	NA
Meaningful change	27–40	2 (1793)	34 (27–40)	1 (1743)	27 (25–29), NA
Directionality of transition scale					
One-sided	9–40	5 (538)	18 (13–21)	3 (383)	15 (8–21), 93%
Two-sided	8–25	19 (3962)	16 (13–20)	18 (3844)	16 (14–19), 91%
Pain scale					
Visual Analogue Scale	8–40	17 (3232)	17 (13–25)	15 (3109)	16 (13–19), 91%
Numeric Rating Scale	10–23	7 (1268)	15 (14–21)	6 (1118)	16 (14–19), 91%
Measurements per patient					
Single	18–40	4 (461)	25 (23–29)	4 (2058)	22 (19–25), 0%
Multiple					
With adjustment	10–18	9 (1104)	15 (13–16)	8 (1031)	15 (13–16), 34%
Without Adjustment/unclear	8–25	11 (2935)	16 (10–21)	11 (2935)	16 (13–20), 95%
Risk of attrition bias					
Low	8–40	8 (1249)	19 (15–25)	8 (1249)	16 (14–18), 81%
High or unclear	9–25	16 (3251)	16 (12–19)	13 (2978)	16 (13–19), 93%
Risk of non-representative sampling					
Low	8–25	6 (1187)	16 (10–21)	6 (1187)	16 (11–21), 96%
High or unclear	9–40	18 (3313)	18 (14–24)	15 (3040)	16 (14–18), 87%

*IQR* inter-quartile range, *ED* Emergency department, *NA* not applicable

^a^The median is based on studies included in the pooled average as well as studies with unavailable standard errors

^b^Total number of patients in studies

^c^I^2^ is the percentage of the variability in results that is due to heterogeneity rather than sampling error (chance); I2 of 0% to 40% might not be important, 30% to 60% may represent moderate heterogeneity, 50% to 90% may represent substantial heterogeneity, and 75% to 100% represents considerable heterogeneity

^d^Includes knee surgery, laparotomy, third molar extraction and mixed surgery (e.g. spinal fusion and splenectomy)

^e^Includes headache, low back pain, sickle cell crisis, rheumatic condition, and pain flare after external beam radiotherapy for bone metastases

### Supplementary outcomes

The supplementary outcomes for pain relief and worsening were only reported from studies using the mean change approach (Table 3). The results showed similarly high heterogeneity. The substantial clinically important difference for pain relief ranged from 18 to 54 mm (23 studies), while minimum and substantial clinically important differences for worsening of pain ranged from 8 to 21 mm increase (18 studies) and from 0 to 66 mm increase (16 studies), respectively.

## Discussion

We included 37 studies (8479 patients) assessing the MCID in acute pain, of which 35 used the mean change approach and seven used the threshold approach. Meta-analyses found considerable heterogeneity between studies and, consequently, no single value of minimum clinical important difference could be meaningfully determined. Study results ranged widely both when they were reported as absolute change (from 8 to 40 mm) and as relative change from baseline (from 13 to 85%). The median of study results based on the mean change approach was 17 (IQR 14 to 23) mm and 23 (IQR 18 to 36) % for absolute and relative values, respectively. Reasons for heterogeneity were explored and baseline pain was identified as a cause of variation in absolute, but not relative, outcomes. In addition, the definition of minimum clinically important change and the use of multiple assessments per patient influenced study results. High heterogeneity was also found for assessments of substantial clinically important difference as well as for worsening of pain.

### Strengths and limitations

To our knowledge, this is the first systematic review of MCIDs in acute pain. We identified 37 studies involving over 8000 patients and a broad range of clinical conditions, study approaches and pain scales. We gained access to unpublished data from 10 studies, including individual patient data from seven studies (918 patients). This ensured high data quality and uniform analysis, and enabled an adequate assessment of the association with baseline pain avoiding the risk of ecological fallacy [24] inherent to aggregated study-level data. The median results of studies providing individual patient data was comparable with the remaining studies and we have no reason to believe these studies were not representative. Association with baseline pain has been reported from individual studies [31, 33, 36, 43, 57–59], but the present review is the first comprehensive assessment of the impact of baseline pain across studies. In addition, we identified variation in study designs (single or multiple assessments) and definitions of patients with minimum relief as factors influencing the MCID.

However, we were not able to fully explain the large heterogeneity among studies. We found no effects of pain scale, but for the comparison of clinical conditions and directionality of transition scale, subgroups involved too few studies to ensure detection of all relevant associations. In addition, our ability to assess clinical condition was limited by the fact that many studies included a mixed patient group and we did not have access to individual patient diagnoses. Similarly, the studies included a variety of analgesia and other treatments which did not allow an assessment of potential impact of interventions. Regarding the risk of attrition bias and non-representative sampling, the majority of studies were categorised as unclear and potential impact could therefore not be assessed. Most importantly, acknowledging the association with baseline pain, it would have been more accurate to base subgroup analyses on relative outcomes, but the data available only allowed comparison of absolute outcomes. We could not assess impact of various description of pain (e.g. “intensity”) or the follow-up time between measurements since there was not enough variation between studies. Furthermore, the available data did not allow an assessment of the potential influence of pre-existing pain level (e.g. if patients are affected by chronic pain in addition to their current episode of acute pain), pre-existing use of pain relief or the psychological state of patients, since this was not reported by any of the studies. Finally, we cannot dismiss the risk of recall bias in studies where patients simultaneously assess their pain status and perceived change [60]. Differences in baseline pain or other methodological or clinical factors may influence subgroup analyses of study-level data. Thus, better access to individual patient data would greatly improve the chances of identifying causes of heterogeneity.

### Other studies

Only few systematic reviews of the minimum clinically relevant change have been published despite a vast number of primary studies. Stauffer [20] and Erdogan [61] reviewed studies of minimum clinically relevant change in pain scales used for chronic rheumatologic conditions, but we have not identified any systematic reviews focusing on acute pain.

The problem of variability in results of studies of minimum clinically relevant change has previously been addressed primarily when attempting to reconcile dissimilar results from different approaches, e.g. anchor-based and distribution-based studies [62]. Our study demonstrates considerable unexplained variation also within anchor-based approaches. In line with our findings, Terwee [63] found variability between results of five studies of minimum clinically important change on the Western Ontario and McMaster University pain subscale for osteoarthritis. In a systematic review of the minimum clinical important difference in chronic pain, we have found similar issues of high study variability (manuscript in preparation).

### Mechanisms and perspectives

We included studies with a patient-reported anchor. While some find that using a patient-reported criterion as an anchor for a patient-reported score is circular and basically flawed [64], we would argue that pain intensity is essentially a subjective experience best expressed by and anchored to those experiencing it. Other observer-based anchors may be used when the outcome of interest is return to work or daily level of activity [65]. The varying content of the patient-reported anchors is, however, problematic. The applied transition scales were either one- or two-sided, allowing patients to report their degree of change (or only relief) by choosing between anywhere from three to 15 response categories. The majority of studies then determined the MCID as a mean change in pain score among everyone reporting a one-category pain relief. However, this value does not apply to all individuals in the group, since their differences in pain are distributed around the mean [14]. In contrast, MCIDs expressed as threshold values are derived with the intention of obtaining the best possible discrimination between patients with and without relevant relief. The frequency of false-positive and false-negative results may be reduced but is not eliminated by this approach. Therefore, caution is always merited when bringing an overall estimate of important change to the level of interpretation for an individual patient [66, 67].

The studies we included varied considerably both in methods and analytical approaches. As would be expected, differences in the definition of patients with minimum important change impacted study results. We also found that the use of multiple measurements per patient resulted in lower outcomes. This corresponded with the finding that outcomes decreased during several follow-ups as patients’ pain declined over time. Furthermore, one in four of the reviewed studies did not differentiate between minimum relief and minimum worsening of pain in their original study reports. The practice of combining groups with minimum change, regardless of its direction, is sometimes based on a seemingly similar distribution of data in the two groups [44]. However, although they may be similar at one point, the MCID for pain relief and worsening will change in opposite directions with variations of baseline pain (since patients with higher baseline pain require larger pain reduction to perceive relief, but smaller increase to perceive worsening of their condition).

The association between MCID and baseline pain may to some extent be explained by ‘regression towards the mean’ as patients are likely to score closer to the mean if their initial scores were more extreme because of chance [68]. However, it is also very plausible that patients with higher pain require greater decrease to perceive relief. Relative changes are therefore more stable indicators of clinically important differences, although they lack interval scale properties at the scale extremes, e.g. when baseline values are close to zero and small degrees of pain change result in very large relative changes [69]. From this review, however, it is clear that the advantage of relative values is largely overlooked, since only 10 of 37 studies (27%) reported relative change.

This review included studies that determined MCID from an anchor-based method using patients’ perception of change to determine clinical importance. Although this is the most common approach, it is just one among a wide range of alternative methods. Revicki noted that retrospective self-reports of pain relief tend to correlate more strongly with the end-level of pain than the start level, implying that the current status matters to patients more than the degree of improvement [70]. This has led to the development of the concept of ‘patient acceptable symptom state’, defined as the level of symptoms which patients feel is acceptable [71, 72]. Patient acceptable symptom state corresponds with the dominant aim of clinical patient care to reduce pain to an acceptable level [73] and could be a strong candidate for an alternative to MCID. Other promising approaches have integrated intervention costs and side effects [74–77].

It is likely that the challenges of MCID, apparent for acute pain, may not be isolated to that specific research area. Acute pain stands out because of the many studies that have been conducted, reflecting the status of acute pain as a core symptom in clinical practice. Our study can thus be seen as a model for a more general challenge with empirical assessments of the MCID.

The methodological challenges embedded in the empirical assessment of MCID are of such an extent that merits caution for its use and interpretation. It is clearly inappropriate to use and interpret MCID as a kind of clinical scale constant – a characteristic which, once empirically determined, is universally valid. This is, however, often the practice seen [78]. Nevertheless, there is a strong and reasonable demand for a structured approach for evaluating whether effects of interventions are clinically meaningful to patients.

### Implications

The choice of a reference value has large consequences for the number of patients needed in a trial, e.g. four times as many patients will be included, if researchers accept a MCID value of 12 mm as compared to 24 mm. Further, the conclusion about the clinical relevance of a trial result is often based on whether a mean difference exceeds a chosen reference value, but with the large span of MCIDs available in the literature, it is highly problematic to randomly pick one or a few single assessments for guidance. The considerable variation means that it is necessary to conscientiously and explicitly reflect on the span of results in relation to context-specific clinical and methodological factors, as presented in this review, with a particular focus of the baseline pain of patients, whether repeated measurements were used, and how minimum relief was defined. A starting point for such an exercise by individual clinicians or researchers, or by consensus building committees, could very well be our overview of studies and their results.

In future studies there is a clear need for uniform guidelines for standardised conduct, analyses and reporting of the MCID, especially for how transition scales and questions are structured and how data are analysed. We strongly encourage using values relative to baseline pain – also for multiple measurements where the patient’s last assessment should be applied as a ‘moving baseline’, standardising the definition of relevant pain relief, and distinguishing clearly between improvement and worsening of pain. In addition, since the influence of clinical and methodological factors is difficult to identify from aggregated data, we encourage improved access to individual patient data to enable further exploration of the causes of heterogeneity.

## Conclusion

The MCID in acute pain varied greatly between studies. Absolute MCID ranged from 8 to 40 mm in 29 studies, and relative values ranged from 13% to 85% in 14 studies. Baseline pain was strongly associated with absolute, but not relative, values and variation in definitions of minimum relief and study designs influenced study results. Due to the heterogeneity between study results, no meaningful overall value of minimum clinically important change can be concluded. Instead, we recommend that MCIDs are considered context-specific and take account of baseline pain. The MCID in acute pain is central for the interpretation of results of randomised trials and meta-analyses and for determining appropriate sample sizes for new trials, but it is potentially misguiding if determined, applied or interpreted inappropriately. Explicit and conscientious reflections on the choice of a MCID value are required, when using it to classify research results as clinically important or trivial.

## Appendix 1

Search strategy

Last search conducted August 2016:

PubMed: (Minimal OR minimally OR minimum OR “clinically significant” OR “clinically important” OR “clinically meaningful” OR “clinically relevant” [Title/Abstract]) AND (Difference OR change OR relief OR reduction [Title/Abstract]) AND ((“Pain Measurement*” OR “Visual Analog Scale”[MeSH Terms]) OR (“numeric rating scale” [Title/abstract])) AND (Pain [MeSH Terms] AND pain [Title]) OR ((correlation AND change AND “visual analog scale” AND “pain relief”[Title]) OR (“minimum clinically significant change” AND pain) OR (“pain intensity ratings” AND “change scores”) OR (“pain assessment tools” AND “acute pain”) OR (“changes in pain intensity correlate with pain relief”) OR (“minimally important difference” AND “pain measurement”) OR (“Minimally important change” AND pain) OR (“minimum clinically important difference” AND pain) OR (“meaningful change” AND pain [title]) OR (“assessing change” AND pain [title]) OR (“minimum clinically important difference” AND “patient-reported outcome”) OR (“detect changes” AND “pain intensity”)).

*Filters: Humans*

EMBASE: ((Minimal or minimally or minimum or clinically significant or clinically important or clinically meaningful or clinically relevant).ti. or (Minimal or minimally or minimum or clinically significant or clinically important or clinically meaningful or clinically relevant).ab. or (Minimal or minimally or minimum or clinically significant or clinically important or clinically meaningful or clinically relevant).kw.) AND ((Difference or change or relief or reduction).ti. or (Difference or change or relief or reduction).ab. or (Difference or change or relief or reduction).kw.) AND ((exp pain assessment/) OR (exp visual analog scale/) OR (exp rating scale/)) AND exp pain/dm, dt, ep, th [Disease Management, Drug Therapy, Epidemiology, Therapy].

*Limit to human, Limit to exclude MEDLINE journals*

Cochrane Library: (Minimal OR minimally OR minimum OR “clinically significant” OR “clinically important” OR “clinically meaningful” OR “clinically relevant”:ti,ab,kw in Cochrane Reviews (Reviews and Protocols), Other Reviews, Trials and Methods Studies) AND (Difference OR change OR relief OR reduction:ti,ab,kw in Cochrane Reviews (Reviews and Protocols), Other Reviews, Trials and Methods Studies) AND (“Pain Measurement” OR “Visual Analog Scale” OR “numeric rating scale”:ti,ab,kw in Cochrane Reviews (Reviews and Protocols), Other Reviews, Trials and Methods Studies) AND (Pain:ti in Cochrane Reviews (Reviews and Protocols), Other Reviews, Trials and Methods Studies) (Word variations have been searched).

## Appendix 2

Data extraction

Descriptive data

For each study we extracted the following data: Publication year, study design, setting, type of intervention, sample size, sex, age and clinical condition of patients, pain level at baseline, type of pain scale (VAS, NRS or other) and scale dimensions, type of anchor including wording of the transition question, concept (e.g. perception of pain relief, overall status or treatment effect), directionality (one- or two-sided), number of discriminating subcategories, wording of items used to obtain minimal clinically important difference, method of data collection including follow-up time, number of follow-up measurements per participant, whether analysis was adjusted for dependency between measurements and applied method, sampling method (e.g. consecutive, random or convenience), attrition rate, and information about any subgroup analysis that had been undertaken in the studies.

Outcome data

For studies assessing clinically important differences using the mean difference approach, we extracted the following outcomes as mean change scores with corresponding standard error or, if unavailable, other measures of variation such as standard deviation or 95% confidence interval:

- The minimal clinically important difference for pain relief (mean absolute and relative change)
- The substantial clinically important difference for pain relief (mean absolute and relative change)
- The minimal clinically important difference for pain worsening (mean absolute and relative change)
- The substantial clinically important difference for pain relief (mean absolute and relative change)

For studies assessing clinically important differences using the threshold approach, we extracted information about the definition of responders (patients with relevant pain relief) and non-responders and the cut-off point that differentiated between them with its corresponding sensitivity and specificity.

## Appendix 3

**Table 5:** Relationship between baseline pain and the minimum clinically important difference^a^

Study	Subgroups by baseline pain, mm	Absolute MCID mm (95% CI)	Relative MCID % (95% CI)
Studies assessing MCID stratified by baseline pain			
Stahmer [55]	≤50 > 50	NR NR	25 (SE: 10)^b^ 35 (SE: 6)^b^
Voepel-Lewis [59]	<50 ≥ 50	0 (−30 to 20) 20 (13 to 25)	NR NR
Bird [31]	<34 34–66 > 66	11 (7 to 15) 17 (13 to 21) 30 (17 to 43)	NR NR NR
Cepeda [33]	Moderate (median: 60) Severe (median: 80)	13 (12 to 14) 18 (17 to 19)	20 (18 to 22) 20 (19 to 22)
Tsze [57]	<40 40–69 ≥ 70	NR NR NR	13 (0 to 29) 16 (10 to 23) 18 (11 to 29)
Tubach [58]	Low (mean: 55) Intermediate (mean: 70) High (mean: 85)	34 (29 to 38)^c^ 45^c^ 63 (54 to 68)^c^	NR NR NR
Studies assessing correlation between MCID and baseline pain			
Barton 2002 (reported in Jensen 2002 [43])	Correlation with baseline pain	0.48 (*P* < 0.001)	0.35 (*P* < 0.01)
Rasmussen 2002 (reported in Jensen 2002 [43])	Correlation with baseline pain	0.52 (*P* < 0.001)	0.34 (*P* < 0.01)
Farrar [36]	Correlation with baseline pain	Patients with higher baseline pain reported greater relief (e.g. 90 vs. 40 mm, *P* = 0.048)	No significant differences in degree of pain relief

^a^Two additional studies are not included, since they did not differentiate between pain relief and worsening when assessing the relationship between baseline pain and MCID (Kelly [46] and Lopez [21])

^b^Patients answering ‘some’ or ‘partial’ relief

^c^Data is 75th percentile among patients answering ‘a great deal better’ (13) or ‘a good deal better’ (14) on a 15 category scale

*MCID* minimum clinically important difference, *NR* not reported, *CI* confidence interval

## Abbreviations

AH: Asbjørn Hróbjartsson
BT: Britta Tendal
EB: Eik Bjerre
JH: Jørgen Hilden
MCID: Minimal clinically important differences
MDH: Maria Damkjær Hansen
MFO: Mette Frahm Olsen
NEL: Nino Emmanuel Landler
NRS: Numerical rating scale
VAS: Visual analogue scale
